# Navigating the System of Regulation and Practice in the UK: Towards a Posthuman Institutional Ethnography of Nursing

**DOI:** 10.1111/jan.17077

**Published:** 2025-06-05

**Authors:** Jamie Brian Smith, Rosie Stenhouse

**Affiliations:** ^1^ Gender in Medicine Charité Universitätsmedizin Berlin Germany; ^2^ Nursing Studies University of Edinburgh Edinburgh UK

**Keywords:** burnout, emotional labour, gender roles, healthcare technology, nursing practice, Posthuman Institutional Ethnography, regulatory frameworks, systemic barriers

## Abstract

**Objective:**

To explore how regulatory frameworks, material constraints, and systemic conditions influence nursing practice, focusing on the Nursing and Midwifery Council (UK) Code, emotional labour, gendered expectations, and healthcare technology.

**Design:**

This qualitative study employed a Posthuman Institutional Ethnography (PIE) approach to understand the material and social dynamics that shape nursing practice.

**Setting(s):**

A renal ward in a large acute National Health Service (NHS) hospital in the UK.

**Participants:**

The sample consisted of 10 practising nurses, aged from their mid‐20s to 50s, with varying lengths of service from 3 to over 30 years, offering diverse perspectives on nursing practice.

**Methods:**

Data were collected from October 2018 to April 2019 through documentary analysis, participatory ethnography, multimedia diaries, and semi‐structured interviews. Thematic analysis, guided by posthuman and new materialist frameworks, examined how human and non‐human actors interact in the production of nurse work.

**Results:**

The Code's emphasis on individual accountability often clashes with systemic barriers such as staffing shortages, outdated healthcare technology, and limited resources, leading to distress and burnout among nurses. Gendered expectations further exacerbate the burden on nurses, contributing to feelings of inadequacy, exhaustion, and emotional strain. Inefficient electronic health records (EHRs) were identified as significant barriers to effective nursing practice.

**Conclusions:**

Addressing systemic barriers is crucial to creating a supportive environment for nurses. Shifting from a model of individual accountability to one of systemic responsibility is vital for enhancing nurse well‐being and improving patient care quality. Policy changes must acknowledge systemic factors such as staffing, technology, and resource availability to create a sustainable and effective healthcare system that supports nursing practice.

**Patient or Public Contribution:**

The study design includes participatory methods where participants create the framing and context of the data included. However, this study did not include patient or public involvement in its design, conduct, or reporting.

**Summary**

- What is already known
  ○ The Code sets high standards for nursing practice, often placing significant pressure on individual nurses.
  ○ Systemic barriers such as staffing shortages, resource constraints, and outdated technology create challenges in adhering to these standards.
  ○ Emotional labour and gendered expectations contribute to distress and burnout among nurses.
- What this paper adds
  ○ Highlights the need to shift from an individual accountability model to systemic responsibility in nursing practice
  ○ Demonstrates how systemic barriers and technological inefficiencies impact nurses' ability to comply with regulatory standards.
  ○ Provides a critical posthuman perspective on the socio‐material factors influencing nursing work, emphasising the distributed nature of care.

## 1 Introduction

The Nursing and Midwifery Council (NMC) Code functions as a central regulatory framework governing nursing practice in the UK. However, the regulatory landscape extends beyond *The Code*, incorporating statutory, legislative, and policy frameworks that shape professional practice. The Health and Social Care Act (2012) and the Care Quality Commission (CQC) regulations play a critical role in defining standards of care, particularly in relation to staffing levels and patient safety. Moreover, guidance from NHS England, the Scottish Government, and the Welsh Government informs workforce planning, emphasising safe staffing and quality assurance measures. While this paper primarily focuses on The Code, it is important to acknowledge these broader legal and regulatory contexts.

## 2 Background

Nursing occupies a pivotal role in healthcare, situated at the intersection of care, politics, and ethics (Benner 1984). Power dynamics, embedded within healthcare institutions, significantly influence not only how nursing is practised but also how it is valued and structured (Allen 2014; Foucault 2003). In this article, we focus specifically on The Code as part of a broader research project on regulatory frameworks in nursing. While there are multiple overlapping policies and regulations influencing nursing practice, this paper examines The Code's impact on individual accountability and systemic challenges, particularly through a posthuman institutional ethnographic lens.

Historically, nursing's professionalisation was shaped by gendered power dynamics. Florence Nightingale framed nursing as an extension of women's caregiving roles, reinforcing its feminised, subordinate status within medicine (Maggs 1996). This hierarchy persists (Gamarnikow 1978; Hopkins‐Walsh et al. 2022). The late 20th‐century Project 2000 sought to elevate nursing through education and credentialing (Aiken et al. 2002, 2013; Allan 1998; Allan et al. 2008), improving patient outcomes while creating new stratifications. However, the focus on technical expertise often devalues emotional and relational labour—key, but harder‐to‐quantify aspects of care (Jackson et al. 2021; Mol 2002; Theodosius 2008).

### 2.1 The Role of Regulation in Nursing Practice

The Code functions as a central regulatory framework governing nursing practice. The Code sets out the professional standards of practice and behaviour for nurses and midwives in the UK, ensuring public trust and accountability (Nursing and Midwifery Council, UK 2015). However, the emphasis on individual responsibility within the Code often places nurses in a difficult position, particularly when systemic barriers such as staffing shortages, high patient loads, and resource limitations prevent them from meeting these regulatory expectations (Smith, Willis, et al. 2024). The Care Quality Commission (CQC) assesses safe staffing levels as part of its regulatory oversight, linking workforce capacity to the delivery of safe and effective care. Additionally, staffing guidance from NHS England, the Scottish Government, and the Welsh Government emphasises the importance of adequate workforce planning to meet patient needs. The misalignment between these regulatory ideals (safe staffing levels) and the material realities of healthcare can lead to significant emotional and ethical challenges for nurses—a phenomenon described elsewhere as ‘The Vitruvian Nurse’ (Smith, Willis, et al. 2024). For example, the Royal College of Nursing (RCN 2022) has reported that 83% of surveyed nurses feel that staffing levels are insufficient to meet patient care demands, highlighting the disjuncture between regulatory expectations and workplace realities.

Feminist scholars have critiqued the institutional frameworks that regulate nursing, arguing that caregiving is often devalued because it is associated with emotional labour—work that is gendered and therefore rendered invisible (Laurin and Martin 2022; Tronto 1993). In healthcare, technical aspects of care are privileged over the relational and emotional dimensions, despite the latter being integral to holistic patient care (Theodosius 2008). This reinforces a hierarchy that marginalises the very elements of nursing that contribute to compassionate, patient‐centred care.

### 2.2 Critical Posthuman Perspectives on Nursing

Critical posthumanism rethinks power in nursing, decentring the human and recognising how non‐human entities co‐produce care. Traditional models prioritise human expertise, particularly physician authority (Braidotti 2019; Greco 2004; Mol 2002; Mol et al. 2015). Posthumanism, however, sees nursing as a socio‐material practice, shaped by interactions between humans, technologies, and environments (Mol 2002; de la Puig Bellacasa 2017). Haraway (2016) concept of “staying with the trouble” and Barad's (2012) “Intra‐action” highlights how care emerges through dynamic human‐non‐human entanglements. This perspective challenges human‐centric models (Latour 1993), viewing care as co‐produced by nurses, patients, technologies, and institutions (Braidotti 2013). Hospital layouts, electronic records, and medical devices influence nursing practice, shaping how care is delivered (Barad 2007; Smith, Klumbytė, et al. 2024; Smith and Willis 2020).

The decision to employ posthuman methods in this research is grounded in the need to explore the complexities of nursing practice beyond human‐centred frameworks. Posthuman approaches, such as those developed by Anne Marie Mol, emphasise the relational and socio‐material aspects of healthcare, allowing us to understand how nursing work is co‐produced by both human and non‐human actors (Mol 2002; Mol et al. 2015). Mol's ethnographic work, particularly her concept of the logic of care, demonstrates how healthcare is an emergent, dynamic practice shaped by multiple interacting elements, including technologies, institutional policies, and embodied experiences.

New materialist and posthuman methodologies provide tools to explore these entanglements, focusing on how materialities—such as medical devices, spatial configurations, and regulatory texts—interact with human actors to shape care practices (Fox 2016, 2017; Fox and Alldred 2016). While posthumanism shares certain resonances with earlier critiques of modernity and human‐technology interactions (Latour 1993), it is not merely a reframing of existing ideas but rather a significant epistemological shift (Braidotti 2013). The emphasis here is on how material‐discursive forces, including regulatory frameworks and cultural‐political structures, are not solely human constructs but emerge through entanglements that exceed human intentionality. This perspective aligns with scholars such as Barad (2007) and Braidotti (2013), who argue that posthumanism fundamentally challenges human exceptionalism and reveals the distributed nature of agency in contemporary healthcare systems.

Using posthuman methods also allows us to challenge traditional notions of accountability and responsibility in healthcare. By recognising the role of non‐human actors, we can shift the focus from individual blame to systemic and relational approaches, highlighting how the conditions of care are produced collectively. This perspective is crucial for developing more sustainable and supportive healthcare environments that acknowledge the complex realities of nursing work.

This paper reports on an aspect of the findings from a larger study focused on understanding the characteristics of nurse work. The focus of this paper is to present findings on how institutions and regulatory frameworks influence the production of nurse work.

## 3 Methods

**FIGURE 1:**
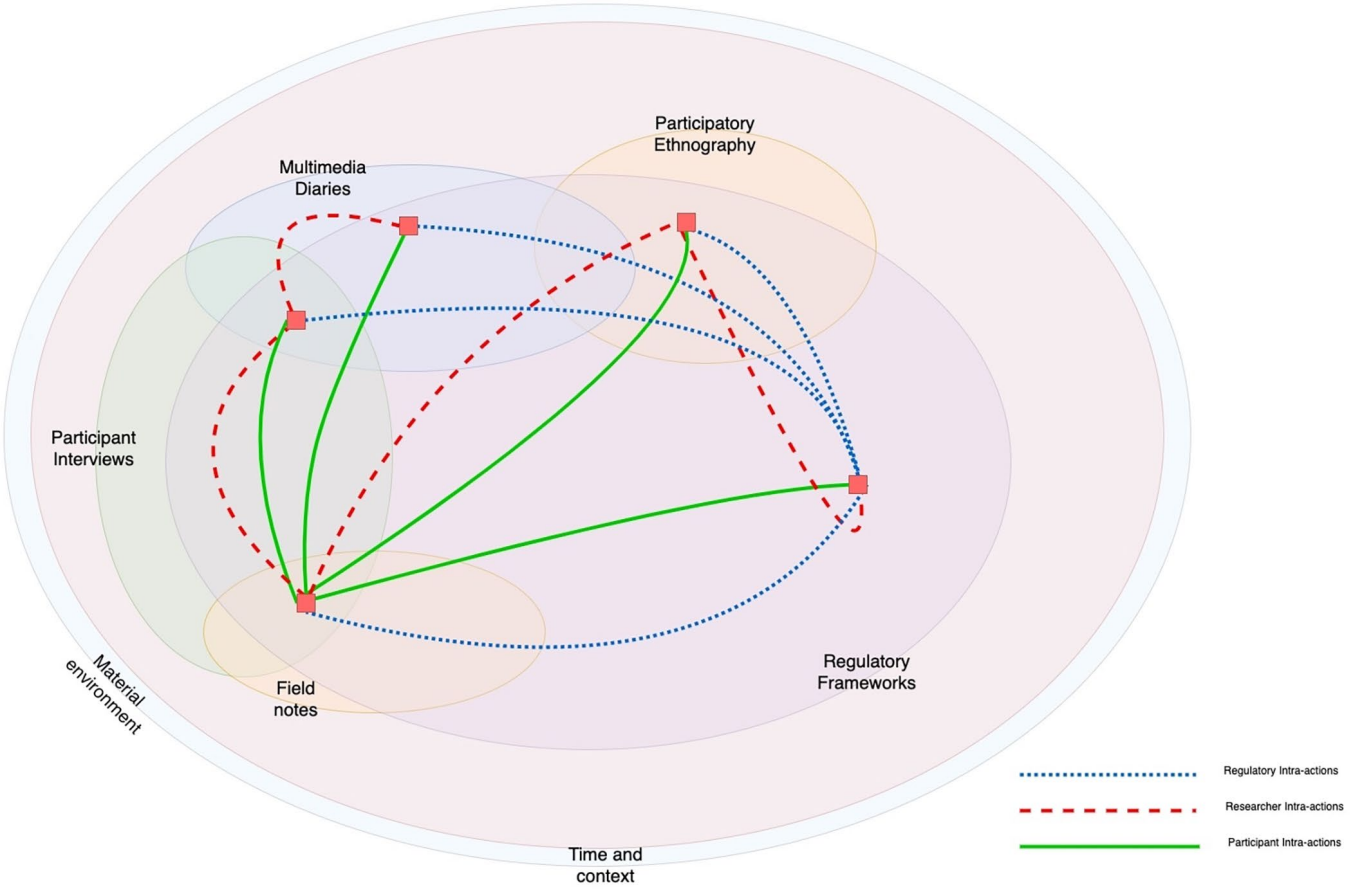
An illustration of data sampling.

This study employed Posthuman Institutional Ethnography (PIE), an extension of traditional Institutional Ethnography (IE) that examines how both human and non‐human elements co‐produce institutional realities. IE focuses on how everyday activities are coordinated by institutional forces (Smith 2005), while PIE incorporates posthumanist and new materialist perspectives to explore interactions between people, places, technologies, and more‐than‐human agents in shaping nurse work and care delivery (Taylor and Fairchild 2020). PIE is particularly suited to this study, as nursing in a large NHS hospital involves complex relations between human actors (nurses, patients, administrators) and non‐human elements (medical technologies, hospital infrastructure, regulatory frameworks). By adopting PIE, this research examines how nursing practices are materially (co)produced through these interactions and shaped by broader regulatory, technological, and social conditions (see Figure 1).

The design was post‐qualitative (St. Pierre 2019, 2021), which challenges traditional humanist assumptions in qualitative research by integrating speculative, affective, and relational dimensions alongside material and discursive realities. This approach extends beyond a representational logic to explore how knowledge is not only observed but also dynamically co‐produced through entanglements of human, non‐human, and imagined elements. By incorporating documentary analysis, participatory ethnography, multimedia diaries, and semi‐structured interviews, this study foregrounds both the tangible and the intangible dimensions of nurse work. Each of these methods allowed for an in‐depth exploration of how nursing practice is shaped and valued within institutional and material assemblages (Doucet and Mauthner 2002; Smith 2005).

### 3.1 Research Setting

The study was conducted at a large acute NHS hospital in the UK over a period of six months. This setting was chosen because of its size and complexity, providing a rich environment in which to examine the diverse forces shaping nurse work. The selection of an acute hospital was deliberate, as these environments are typically governed by strict regulatory standards (e.g., Care Quality Commission, the NMC Code of Conduct), involve the extensive use of medical technology, and represent a nexus where care is negotiated between numerous human and non‐human actors (de la Puig Bellacasa 2017).

### 3.2 Sampling

Participants were recruited using a purposive sampling strategy (Patton 2002), selecting nurses from a renal ward in a large acute hospital in the UK. Data were collected from October 2018 to April 2019. Participants were approached through a combination of email invitations, informational posters in break rooms, and personal referrals; this paper reports on an aspect of the findings from a larger study focused on understanding the characteristics of nurse work.

The final sample consisted of 10 practising nurses, selected for their diverse range of experiences and roles within the hospital. The participants ranged in age from mid‐20s to 50s and included both male and female nurses. Their length of service varied from 3 years to over 30 years, providing a breadth of perspectives on how nursing work is structured and valued (Creswell and Poth 2016). No explicit refusals to participate were noted.

### 3.3 Documentary Analysis

A key component of the PIE methodology involves examining the institutional texts that shape and regulate work practices (Smith 2005; Taylor and Fairchild 2020). For this study, the primary document analysed was the NMC Code of Conduct, the regulatory framework governing nursing practice in the UK. The analysis focused on identifying the ways in which the Code prescribes nursing practice, and how these prescriptions are negotiated in the everyday realities of nurse work (Mol 2002; Smith and Willis 2020). In addition to the Code, other documents such as internal hospital policies on care delivery, job descriptions, and NHS guidelines were analysed to understand how institutional texts coordinate nursing activities (Coffey and Atkinson 1996; Silverman 2010).

### 3.4 Participatory Ethnography

Participatory ethnography was used to explore the lived realities of nurse work in the hospital setting (Hammersley 2018; Hammersley and Atkinson 2019). The primary author (JS) spent three months observing nurses in one acute care ward, participating in their everyday activities, and taking detailed field notes on the material and relational aspects of their work. The primary researchers' role was as a participant‐observer, where they engaged in informal conversations with nurses during their shifts and observed their interactions with patients, medical technologies, computer systems, patient records, and other staff (Pink 2009). Field notes were taken throughout the observations, focusing on the embodied and material practices of care and the ways nurses navigated the institutional and regulatory constraints of their work.

### 3.5 Multimedia Diaries

To acknowledge the material and embodied dimensions of nurse work beyond what could be observed in a limited timeframe, participants were asked to keep multimedia diaries over a three‐week period (Rose 2012, 2022). Participants used smartphones and were encouraged to take photographs, videos, and voice recordings that illustrated their daily work practices. Participants were provided with a prompt sheet to support structuring their entries, which included:

- How are you?
- How is your day going?
- What has been the best part of your day so far?
- What has been the most challenging part of your day?

This method allowed nurses to document moments that they found particularly meaningful or challenging in their day‐to‐day work, offering a visual and auditory supplement to the ethnographic data (Pink 2020). The multimedia diaries provided insight into how nurses experience and negotiate the materiality of their work environments. For example, photographs of cluttered medication rooms or videos of interactions with malfunctioning equipment highlighted the non‐human elements that shape nurse work in ways that might be overlooked in traditional ethnography (Fox 2016).

### 3.6 Semi‐Structured Interviews

Semi‐structured interviews were conducted with all participants at the end of the multimedia diary period (Kvale and Brinkmann 2009). The information from participants' multimedia diaries was used as entry points to data analysis and to create interview schedules. The multimedia diaries were analysed thematically and guided by posthumanist and new materialist frameworks (Fox and Alldred 2022). This analysis produced significant events or ideas that were then used to produce individual interview schedules. These interviews lasted between 45 min and 1 h and were designed to explore the participants' reflections on their work, the regulatory environment, and the material and relational challenges they encountered. The interviews were audio‐recorded and transcribed verbatim.

### 3.7 Data Analysis

**FIGURE 2:**
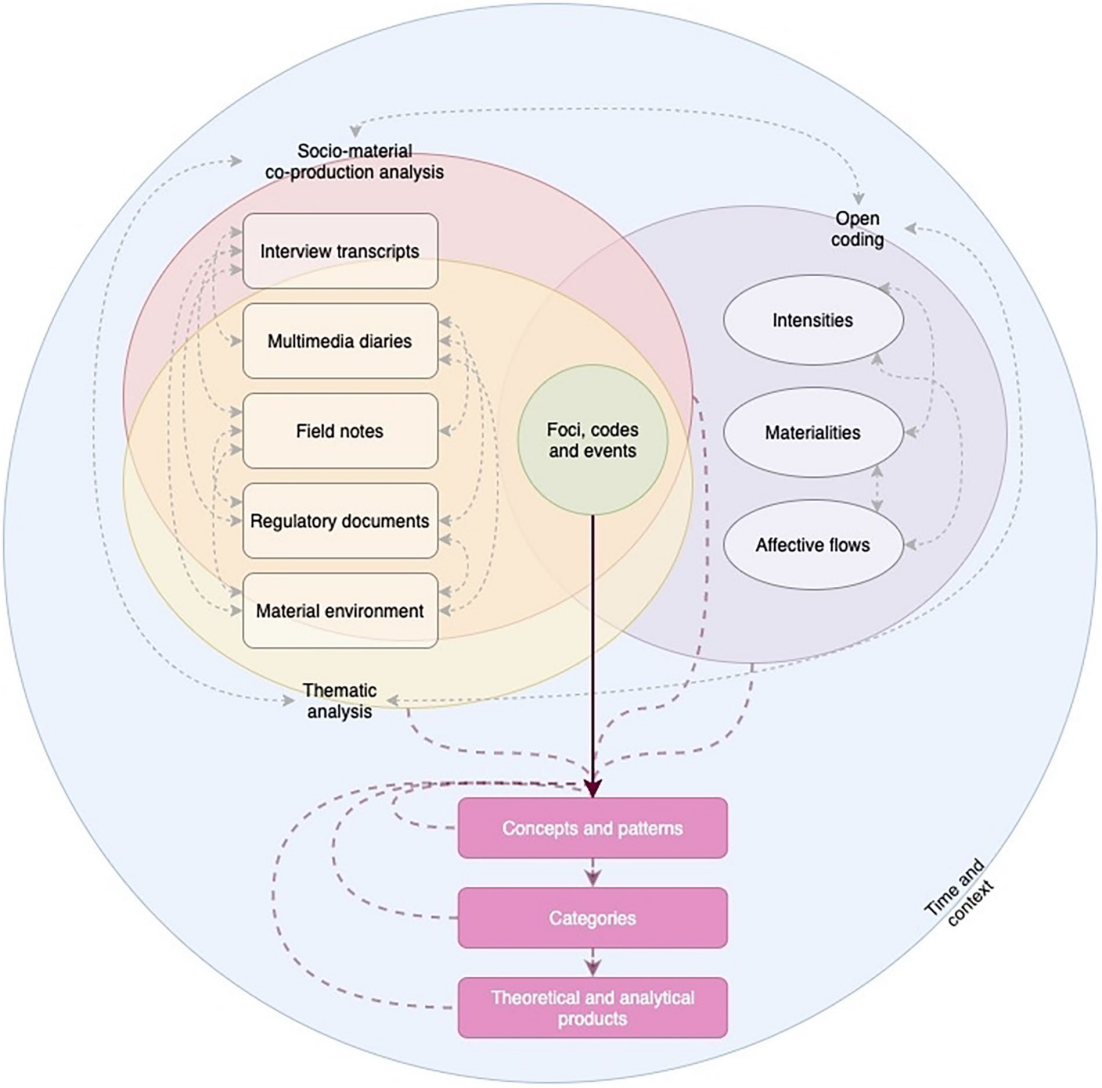
A schematic of data analysis with a new materialist perspective.

Data analysis followed an affective thematic approach, guided by posthumanist and new materialist frameworks, emphasising the role of non‐human actors and materialities in shaping nursing practice (Fox and Alldred 2022; Mol 2002; de la Puig Bellacasa 2017; Taylor and Fairchild 2020). The transcripts were then analysed thematically, focusing on how participants articulated their experiences of institutional constraints, material challenges, and their interactions with patients and colleagues (Braun et al. 2019). Drawing on Fox and Alldred's (2022) posthuman and new materialist methodology, the analysis moved beyond human‐centric perspectives to consider how affect, materialities, and discourses co‐produce nurse work. Analysis was conducted and confirmed by both researchers. MAXQDA (VERBI Software 2023) data analysis software was used for organising and coding data. See Figure 2 for a data analysis schematic.

### 3.8 Coding Process

**FIGURE A1:**
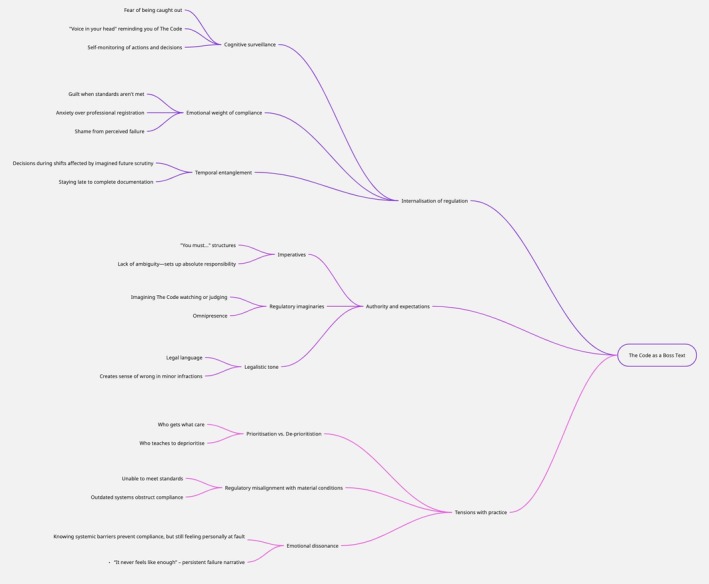
Example of a coding tree.

The initial stage involved open coding of interviews, multimedia diaries, and field notes for themes produced, including interactions with non‐human actors such as medical technologies and clinical spaces. Using Fox and Alldred's focus on “affective flows” and “intensities,” the analysis identified affective engagement (e.g., frustration with malfunctioning equipment) and sensory experiences as key components in the material co‐production of care. An example of a coding tree from this process is available in Figure A1.

### 3.9 Mapping Assemblages

In line with assemblage thinking (Deleuze and Guattari 1988), the analysis mapped the relational networks of human and non‐human actors. Care practices were viewed as the product of these assemblages, where agency was distributed across bodies, technologies, and spaces (Fox and Alldred 2022; de la Puig Bellacasa 2017). For instance, a nurse's interaction with a dialysis machine was understood not as a one‐way process but as an interdependent relation that included the machine, room layout, and institutional regulations (see Figure 2).

### 3.10 Discourse and Materiality

The analysis integrated discourse and materiality (Fairclough 1992), examining how regulatory frameworks like The Code shaped nurse work while interacting with material realities. Barad's (2007) the concept of agential realism informed this process, revealing how material and discursive forces co‐construct nursing practices. For example, institutional policies and physical spaces worked together to influence how care was delivered.

### 3.11 Reflexive and Iterative Analysis

Data collection and analysis occurred in tandem, incorporating Walsh's (2024) framework of personal, interpersonal, methodological, and contextual reflexivity. Interpersonal reflexivity considers relational dynamics, acknowledging the primary researcher's dual role as a researcher and practising nurse. Methodological reflexivity ensured transparency in how research design shaped findings, while contextual reflexivity addressed broader socio‐political and regulatory influences.

As the primary researcher was a nurse also practising in the clinical area, observations were documented only for consenting participants, with all data anonymised and non‐anonymisable details omitted. Patient data were excluded, and nurses were reminded of their professional responsibilities, including confidentiality and the potential for data publication. The primary researcher's privileged access to information was acknowledged, and ethical considerations guided decisions on data inclusion. These issues were discussed regularly and extensively within the research team to ensure research integrity. To address concerns about professional accountability and regulatory scrutiny, participants were assured of data anonymity, and systemic issues discussed were not used to identify individuals. This aligns with Walsh's (2024) emphasis on critically engaging with researcher‐participant power dynamics. Ethical approval ensured transparency, and participants had the right to withdraw.

Reflexive strategies included maintaining a research journal, engaging in peer discussions, and seeking contradictory evidence within the data (Walsh 2024). These measures problematised assumptions, ensuring a nuanced interpretation that did not reduce nursing experiences to a singular critique of regulation. This iterative and reflexive engagement reinforced the study's post‐qualitative orientation, attending to the speculative, emergent, and relational dimensions of inquiry.

### 3.12 Ethical Considerations

Ethical approval was obtained from both the University of Edinburgh ethics committee (REC: NURS038, 2018) and the NHS Lothian internal ethical review board (REC No: 18/NRS/003, 2018). Participants were provided with detailed information about the study, including assurances of confidentiality and the right to withdraw at any time. All names and identifying details are anonymised and pseudonyms used, and care was taken to ensure that the multimedia data (e.g., photographs and videos) did not reveal the identities of patients or staff (British Sociological Association 2017).

The study involved the use of multimedia diaries, which may have added an additional burden on participants, particularly given the already demanding nature of their workload. To mitigate this potential burden, participants were offered flexible timelines for diary entries and were encouraged to document their experiences at times that were convenient for them. They were also reminded that their participation in this part of the study was voluntary, and they could decide how much or how little they contributed. Furthermore, emotional support was made available for participants who found reflecting on their daily work distressing.

Anonymisation of data was a critical aspect of the research process. All multimedia content, including photographs and videos, was carefully reviewed to remove any identifiable information before analysis. In cases where patient information was captured inadvertently, these details were edited out, or the content was excluded from the study entirely to maintain confidentiality. Audio recordings were transcribed, and all personal identifiers were removed during the transcription process to ensure anonymity. The anonymisation procedures followed the ethical guidelines set out by the British Sociological Association (2017) and adhered to data protection regulations. The researchers took care to maintain a collaborative approach with participants, ensuring that their contributions were valued and respected throughout the study. Participants were given opportunities to review and provide feedback on the findings related to their data, supporting a co‐constructive approach to the research.

## 4 Findings

The findings of this study are based on data from field notes, multimedia diaries, documentary analysis of The Code, and participant interviews. No explicit reference was made to The Code during recruitment or initial data collection. However, during participatory ethnography and multimedia diaries, nurses repeatedly invoked The Code as a structuring force in their decision‐making, emotional labour, and documentation practices. The mean age of participants was 35.3 years, and their mean length of working as a nurse was 8 years. The analysis produced foci on The Code as a *Boss Text* and Compliance in Practice. The findings highlight the complex interplay between regulatory expectations, material conditions, and the challenges faced by nurses in their daily work.

### 4.1 The NMC Code as a Boss Text

**FIGURE 3:**
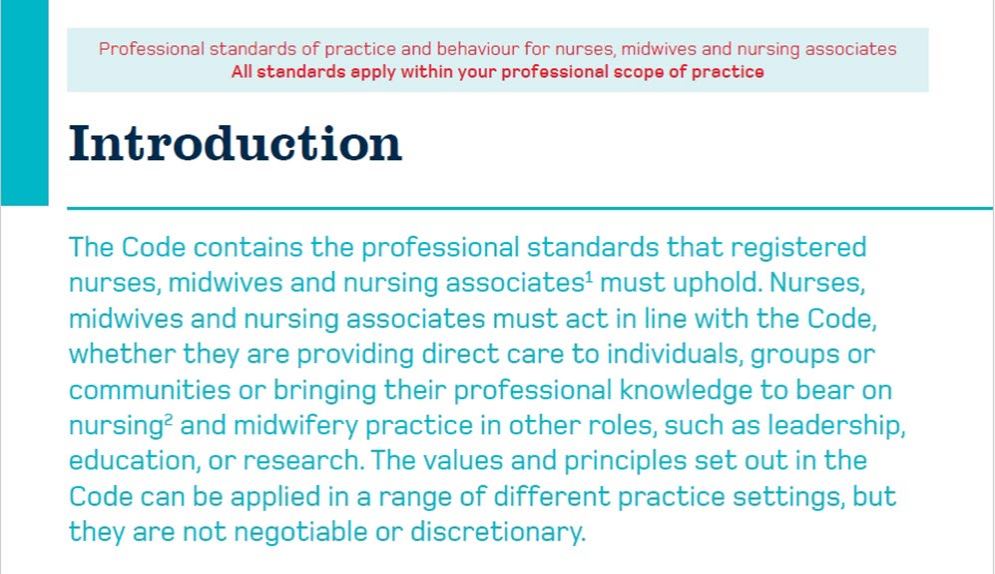
Page 3, The Code, NMC (2015).

The Code functions as a central regulatory text that shapes nursing practices, acting as a boss text (Smith 2005) that influences both the practical and emotional aspects of nursing work. The findings illustrate how the Code's requirements intersect with systemic constraints, creating tensions between regulatory compliance and the socio‐material realities of care delivery. Participants consistently described the characteristics of the Code in line with the function of the Code as a ‘boss text’, highlighting its authoritative nature that dictates standards for care. Its authoritative language, including imperatives like “*You must*,” establishes clear directives for nurses (see Figure 3).

One participant reflected in her multimedia diary that she didn't feel like she had been able to “do the care” that she wanted to during a shift, when prompted about this in her interview she gave more context saying “We are always thinking about the Code. Every decision has to align with those standards, or it comes back to bite you.” (*Participant 2*). This encapsulates the pervasive influence of the Code on decision‐making and the perceived consequences of non‐compliance. The emphasis on public trust and professional accountability adds pressure on nurses, as they constantly evaluate themselves against these ideals. One nurse shared, “It's like you have this voice in your head reminding you of the Code. Every decision you make has to pass that test.” (*Participant 7*). This sentiment was echoed across the multimedia diaries, where participants documented moments of decision‐making, often linking them explicitly to The Code's expectations. This internalisation adds a cognitive burden as nurses self‐monitor to align their actions with regulatory expectations.

### 4.2 Individual Responsibility vs. Systemic Barriers

The Code emphasises individual accountability, positioning nurses as solely responsible for maintaining high standards of care. However, our findings reveal systemic barriers that often prevent nurses from meeting these expectations. Limited staffing, high patient loads, and resource shortages were frequently cited as obstacles. One of the participants had a student nurse working with her, and she noted in her diary that she had been “teaching them to prioritise”. When reminded of this in a longer interview, she stated, “The Code says I should always provide the best care, but how do I do that when I've got five patients and one of them is critical? I can't be everywhere at once” (*Participant 7*). This day had also been noted in the field notes as conflict between prioritisation and de‐prioritisation, highlighting a conflict between regulatory ideals and the realities of practice, which creates a sense of inadequacy among nurses. This sense of inadequacy is vividly illustrated by a diary entry from one participant who wrote:

I felt completely overwhelmed today. The Code expects us to be everywhere at once, but with limited staff, it's impossible. I had to make a choice between attending to a patient in pain or completing my documentation, and either way, I felt like I was failing. (Participant 8)

**FIGURE 4:**
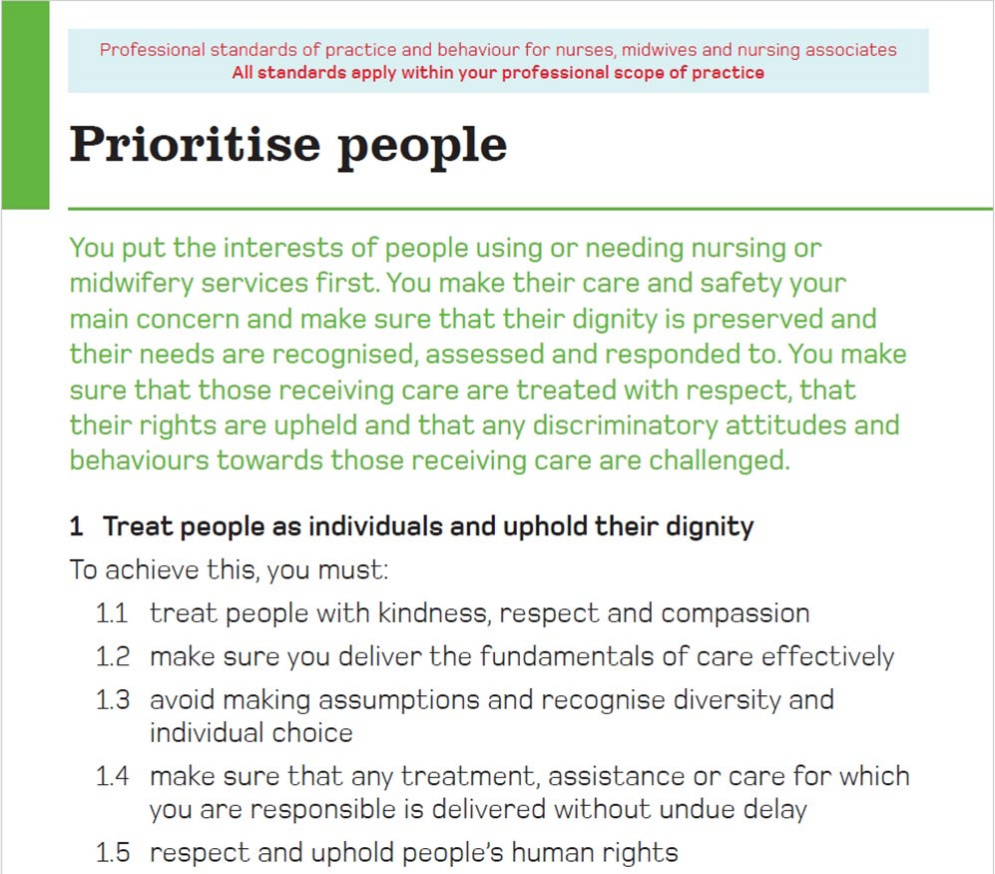
Page 6, The Code, NMC (2015).

This example shows the emotional impact of striving to meet expectations amidst systemic constraints. The Code instructs nurses to “prioritise people” and put patients at the centre of their practice (see Figure 4), but time constraints and workload pressures often force difficult choices about who receives attention. One diary entry illustrated this dilemma: “If someone in Bed 3 is having chest pain, I'm not going to make the man in Bed 1 a cup of tea, even though he's been asking for it all morning.” (*Participant 5, Female, 42*). This can be juxtaposed with another participant saying in their interview, “They (The NMC) tell us how to prioritise, but it doesn't say how it will back us up when we have to deprioritise” (*Participant 6*).

The Code as a boss text was produced consistently across both the field notes and the multimedia diaries. In field observations, it was clear that nurses often faced critical moments where systemic barriers made it challenging to meet The Code's standards. Multimedia diaries further documented participants' frustration, highlighting moments where they had to choose between patient needs, such as providing comfort or completing necessary documentation. For example, one participant recorded a diary entry about a particularly hectic shift where they had to decide between sitting with a distressed patient or catching up on overdue medication records. The participant reflected, “It was heartbreaking to leave them but I'd been in there all day and needed to crack on” (*Participant 4*). This example highlights the difficult decisions nurses face, demonstrating the emotional and professional conflict inherent in their roles. This triangulation strengthens the argument that systemic barriers fundamentally shape the ability of nurses to meet regulatory standards.

These examples of The Code in practice demonstrate that while nurses are aware that systemic barriers limit their ability to comply with The Code, they also internalise the sense of failure when unable to meet its standards. This internalisation contributes to an emotional burden that includes a fluid perception of accountability—where systemic failures are perceived as personal shortcomings. Addressing these fluid perceptions of accountability and distinguishing between systemic versus personal responsibility is critical in mitigating the negative emotional impacts on nursing staff and fostering a supportive work environment that acknowledges these complexities.

### 4.3 Consequences of Non‐Compliance and the Emotional Influence

The consequences of failing to meet The Code's standards are both professional and emotional. Participants described feeling anxious about potential repercussions, particularly regarding documentation and patient safety. One nurse explained, “I had a patient who needed care immediately, and I was scared not to document everything else I'd forget. By the time I finished writing everything, I was already running behind with other patients.” (*Participant 9*). This highlights multiple layers of emotional and affective labour as the participant is potentially scared not to deliver the care he wants to, and how time‐consuming documentation can create delays, ultimately impacting patient care.

Many participants described feeling as though they were “not doing enough,” despite working to their capacity. The Code's directive to “act without delay if you believe there is a risk to patient safety or public protection” adds urgency to already strained conditions. One nurse reflected, “You always feel like you're not doing enough, even though you're doing everything you can.” (*Participant 1*). This sentiment was echoed in multimedia diary entries, where participants documented moments of feeling overwhelmed. One participant recorded a voice note stating, “I left work today feeling like I'd let everyone down. We were short‐staffed again… It never feels like enough.” (*Participant 3*). Another nurse shared an image in their diary of a telephone, captioned, “I can still hear it ringing.” These examples illustrate how nurses' distress extends beyond their shift, reinforcing how The Code's demands might contribute to a pervasive sense of personal failure. This sense of inadequacy underscores the emotional labour involved in navigating compliance amidst critical care overlaid by systemic challenges, as nurses are often left feeling that their best efforts are insufficient due to systemic barriers beyond their control.

Nurses would often stay late to finish documenting, ensuring their records were complete despite exhaustion and ongoing technical difficulties with electronic systems. One participant stated, ‘I should have left half an hour ago, but I'll get the next bus’ (*Participant 8*), and is observed in field notes as she sat at a computer typing and looking visibly sweaty and tired. Many nurses described fighting with slow and outdated computer systems, which added to their frustration. One nurse described waiting for the system to load, saying, “By the time the computer lets me log in, I could have already been with my next patient.” (*Participant 5*). This moment encapsulates how nurses experience regulatory pressure not only in their actions but also in their self‐monitoring practices.

The internalisation of The Code's expectations places an individual burden on nurses, even when they recognise that the barriers they face are systemic. This dynamic creates a situation where systemic failures are perceived as individual shortcomings, resulting in a fluid perception of accountability. Nurses may intellectually understand that staffing shortages or resource constraints are systemic issues, but they still feel personally inadequate when unable to meet The Code's standards. This dual burden—recognising systemic issues while internalising responsibility—contributes to significant emotional strain. Emotional regulation becomes challenging as nurses try to balance the awareness of systemic barriers with their internalised expectations of professional competence.

The internalisation of accountability affects how nurses perceive their role and responsibilities, often leading to an unrealistic sense of personal failure. This ongoing struggle highlights the need for healthcare institutions to distinguish more clearly between systemic accountability and individual responsibility. By doing so, they can support nurses in understanding that their perceived inadequacies are often the result of systemic problems, not personal failings. Addressing these dynamics is crucial for reducing emotional exhaustion and promoting more sustainable working conditions.

### 4.4 Compliance in Practice: Navigating Documentation and Direct Patient Care

Building upon the examination of The Code's role in shaping nursing practice, this section explores how compliance manifests in everyday nursing practice, focusing on the challenges of balancing documentation with direct patient care. The Code places significant emphasis on accurate record‐keeping and timely action, yet the realities of practice reveal deep‐seated tensions between these regulatory expectations and the material and emotional demands of patient care.

### 4.5 The Cognitive and Emotional Burden of Compliance

The need for comprehensive documentation, as stipulated by The Code, creates a substantial cognitive and emotional burden for nurses. This is understandably necessary to ensure patient safety and accountability. Participants described how recording every aspect of patient care is not only time‐consuming but also mentally exhausting. One nurse explained, “Sometimes it takes longer to document care than to actually provide it.”(*Participant 6*). This highlights the inefficiency of existing electronic health record (EHR) systems, which compounds the difficulty of meeting The Code's documentation requirements. It was noted from field notes that staff would often be waiting to use computers due to all available workstations being occupied. When computers were available, one participant noted that it could sometimes take a protracted time for the EHR to load “…I log in to the system and then go make my cuppa tea and then come back and do my writing…” (*Participant 5*). These potential barriers in accessing EHR systems compound the difficulty for nurses to meet the documentation requirements expected by The Code. It highlights the role of outdated or cumbersome systems in adding stress and taking time away from patient care, making it challenging for nurses to comply effectively with regulatory standards. This inefficiency means nurses must spend more time on administrative tasks rather than direct patient care, thus increasing their workload and stress levels.

The cognitive burden of documentation is further exacerbated by the need to ensure that all records are clear, accurate, and in line with the Code's standards. Participants spoke about the mental strain of constantly checking and re‐checking their notes to avoid errors that could have serious professional consequences. Field notes captured a nurse during a busy shift who was visibly anxious as they double‐checked medication charts, stating, ‘I can't afford to make a mistake here—if anything goes wrong, it's my PIN (nursing licence) on the line’ (*Participant 10*). One nurse described, ‘You're always second‐guessing yourself when it comes to documentation. It's not just about writing what you did—it's about writing it in a way that would stand up to scrutiny if something went wrong.’ (*Participant 3*). This vigilance reflects the weight of accountability that documentation carries, adding a layer of stress to an existing context of competing demands.

### 4.6 Documentation vs. Direct Patient Care: A Balancing Act

The Code's dual emphasis on patient‐centred care and thorough documentation places nurses in a difficult position, often forcing them to prioritise one at the expense of the other. Many participants expressed frustration at the time spent documenting, which they felt took away from direct patient care. One nurse shared:

I had a patient who was in pain, but I also had to finish documenting the morning meds and I didn't notice quickly and he didn't buzz. By the time I got back to him, he was even more distressed. It feels like the paperwork comes before the people sometimes. (Participant 2)

Balancing documentation and patient care illustrates the systemic disconnect between regulatory standards and the realities of clinical practice. The expectation that nurses can seamlessly integrate thorough record‐keeping with attentive, patient‐centred care fails to account for the pressures of understaffed wards and high patient acuity. One diary entry poignantly captured this tension: “If I focus on writing everything down, I feel like I'm neglecting the person in front of me. But if I skip the documentation, I'm putting myself at risk.” (*Participant 4*). This dilemma underscores the emotional and ethical challenges nurses face in their efforts to comply with the Code.

## 5 Discussion

This study provides insight into the intricate and multi‐dimensional challenges inherent in nursing practice, particularly as shaped by regulatory frameworks like The Code, which, while specific to the UK, reflects challenges likely mirrored in other healthcare systems globally. However, it is important to acknowledge that this study offers a piece of the broader puzzle rather than a comprehensive account. Using a critical posthuman and new materialist perspective, we make perceptible how nursing is not a linear activity but rather a socio‐material practice intricately linked to systemic constraints, regulatory demands, and gendered assumptions. This approach allows for a more nuanced understanding of how systemic and material realities shape the care nurses provide and their experiences within healthcare environments.

### 5.1 Systemic Constraints and Regulatory Realities

The findings illustrate that nurses internalise The Code to such an extent that it functions as a persistent regulatory force, shaping their actions even when not consciously considered. This is reinforced by the emphasis on individual accountability, which holds nurses personally responsible for care outcomes despite systemic constraints. Aligning with broader critiques of regulatory governance (Kennedy et al. 2015), this focus on professional accountability often prioritises compliance over systemic responsibility for safe, effective, and equitable care. As The Code positions individual nurses as the primary locus of responsibility, they remain acutely aware that their professional standing hinges on adherence to its directives. Consequently, rather than serving as complementary frameworks, regulatory standards frequently overshadow other decision‐making considerations, such as evidence‐based practice, patient needs, and health equity. The literature on institutional ethnography provides valuable comparative insights. Rankin's (2017) work on materiality and documentation highlights how regulatory frameworks intersect with healthcare technologies to shape nursing practice, often adding burdens rather than facilitating care. Similarly, studies on nurse call systems and medication management have demonstrated how technological infrastructures interfere with nursing work rather than support it (Folkmann and Rankin 2010; Rankin and Campbell 2009; Harvey et al. 2018; Klemets 2016).

A critical posthuman perspective provides a valuable lens for exploring how nursing is co‐produced through the interactions of human and non‐human actors. This study's findings illustrate how material elements, such as electronic health records, mediate and shape care delivery. For instance, while the electronic health record supports accountability and institutional priorities, it also becomes a site of tension, reflecting the logistical and emotional demands placed on nursing staff. The inefficiencies of EHR systems exemplify the material constraints that hinder compliance and detract from patient‐centred care. However, these inefficiencies are not merely technical flaws but are embedded within broader institutional logics that govern nursing work. Participants' narratives underscore how outdated or cumbersome technologies not only increase workload but also create cognitive and emotional strain, further complicating the balance between documentation and direct care. EHR systems do not just passively constrain practice; they actively structure nursing labour by dictating workflows, prioritising certain tasks over others, and reinforcing managerialist imperatives of accountability, efficiency, and risk management. From a new public management perspective, these systems function as mechanisms of institutional control, ensuring that nursing work aligns with organisational priorities—often at the expense of professional autonomy and relational aspects of care. This aligns with wider neo‐liberal currents in healthcare of over‐emphasising individual responsibility (Rankin and Campbell 2009). By recognising these materialities as active participants in the co‐production of care, healthcare organisations can adopt more inclusive approaches to technology design and implementation, ensuring that tools support rather than hinder nursing practice (Overton 2019; Henderson et al. 2017).

The emotional labour inherent in nursing practice emerges as a central theme in this study, intricately tied to the ethical complexity of care. Participants frequently described the tension between professional expectations and the relational demands of patient care, where systemic constraints often force compromises. This aligns with existing research on moral distress and burnout, which identifies emotional labour as both a cornerstone of nursing and a source of significant strain (Smith 2005; Maslach and Leiter 2022). The emotional burden is further exacerbated by The Code's emphasis on accountability, which amplifies feelings of inadequacy when systemic barriers prevent nurses from meeting professional standards. For instance, the expectation to “prioritise people” often clashes with the practical realities of high patient loads and insufficient staffing, creating situations where nurses must make ethically fraught decisions about who receives care. These findings highlight the need for institutional policies that prioritise emotional resilience and provide mental health resources, peer support, and a culture of compassion (Traynor 2017). These findings also call for future versions of nursing practice frameworks to understand the characteristics and implications of a boss text in the context of a regulated and scrutinised profession.

To enhance the relevance and impact of these findings, it is useful to situate them within an international policy context. Regulatory frameworks in different countries offer varied approaches to balancing accountability with systemic support. For example, Scandinavian healthcare models emphasise collective responsibility, where nurses are supported through robust staffing policies and institutional backing rather than being individually held to account for systemic failures (Nordic Council 2015). Similarly, Australia's National Safety and Quality Health Service (NSQHS) Standards incorporate mechanisms to ensure that regulatory expectations are matched with organisational support (Australian Commission on Safety and Quality in Health Care 2021).

Comparing these models to the UK highlights potential areas for improvement. The UK's emphasis on individual nurse accountability, without corresponding systemic investment, places undue stress on practitioners. Drawing lessons from international frameworks that integrate systemic responsibility into regulatory mechanisms could help inform policy reforms in the UK. A shift towards a more balanced approach—where regulatory frameworks acknowledge and address material constraints—could alleviate some of the cognitive and emotional burdens on nurses, enabling them to provide high‐quality care without compromising their own well‐being.

### 5.2 Limitations

This study provides valuable insights into the interplay between regulatory frameworks, systemic barriers, and nursing practice, but several limitations must be acknowledged. The relatively small sample size, drawn from specific UK healthcare settings, may not capture the full diversity of nursing experiences. As a result, the findings may be more reflective of these particular contexts rather than broadly generalisable. Future research should include a larger, more diverse sample to enhance applicability across different healthcare systems.

Participant self‐selection bias is another limitation, as those with strong opinions on regulatory frameworks and systemic challenges may have been more likely to participate, potentially skewing the findings. More stratified sampling could help address this. Additionally, as a post‐qualitative study, the findings are shaped by participants' narratives and researchers' interpretations, introducing an element of subjectivity. A mixed‐methods approach could provide a more balanced and objective perspective.

The study also reflects a specific period, yet regulatory frameworks and healthcare practices evolve. Longitudinal research could explore these changes over time. Despite these limitations, this study contributes important insights into the challenges nurses face in navigating regulatory frameworks and systemic barriers, informing future research and policy development.

## 6 Conclusion

This study highlights the tensions between the Nursing and Midwifery Council (UK) Code's regulatory expectations and the material realities of nursing practice, demonstrating how nurses internalise individual accountability despite systemic barriers. While the Code seeks to ensure professional standards, its structure reflects broader ideological forces—historical, economic, and managerial—that shape how nursing is regulated. Rather than a neutral framework, the Code is a political and discursive artefact that tends towards individual responsibility for nurses while overlooking the structural conditions that constrain care.

By interrogating why the system is as it is, we reveal contradictions that are not incidental but structurally necessary. Nursing is expected to be both compassionate and efficient, autonomous yet tightly regulated. These tensions emerge from neoliberal healthcare models that prioritise cost‐cutting and risk management, embedding audit cultures and digital surveillance into daily practice. The Code operates as a boss text, directing nurses' actions while rendering systemic limitations invisible. Technologies such as electronic health records further entangle compliance with documentation burdens, shaping nursing labour in ways that extend regulatory control.

A posthuman perspective allows us to see regulation as more than a human‐led and objective process, instead considering the entanglements of policies, infrastructures, and care practices. If regulatory frameworks are to be effective, they must shift from enforcing individual responsibility to embracing systemic accountability. International comparisons suggest alternative models that balance professional autonomy with institutional support. Moving forward, we call for a reimagining of regulation—one that does not simply impose compliance but fosters care for both patients and practitioners. This requires a paradigm shift in how nursing work is valued, ensuring that regulatory frameworks reflect the realities of practice rather than reinforcing the unattainable ideal of the Vitruvian Nurse.

## Author Contributions

Jamie B. Smith conducted and wrote up the research described in this paper. Rosie Stenhouse provided consultancy, guidance, and editorial support throughout.

## Conflicts of Interest

The authors declare no conflicts of interest.

## Supporting information


Data S1.

## Data Availability

The data that support the findings of this study are available from the corresponding author upon reasonable request.
